# Welcome to *Mobile DNA*

**DOI:** 10.1186/1759-8753-1-1

**Published:** 2010-01-25

**Authors:** Nancy L Craig, Thomas H Eickbush, Daniel F Voytas

**Affiliations:** 1 Department of Molecular Biology & Genetics, Johns Hopkins University School of Medicine, Baltimore, Maryland, USA; 2 Department of Biology, University of Rochester River Campus, Rochester, New York, USA; 3 Center for Genome Engineering, Department of Genetics, Cell Biology and Development, University of Minnesota, Minneapolis, Minnesota, USA

## Welcome to *Mobile DNA*

We are pleased to launch a new journal, *Mobile DNA*, at a time rich with opportunity for the study of mobile genetic elements. For more than a decade, our discipline has been well fed by data from the various genome sequencing efforts - data that continues to be generated at accelerating rates. Genome sequences have revealed novel element lineages that move by yet unknown mechanisms, providing subject matter for study by molecular biologists, biochemists and structural biologists. In species throughout the tree of life, genome sequences make evident the significant impact mobile elements have on genome organization, by creating genome rearrangements, mobilizing gene fragments, and reshaping the epigenetic landscape. At a mechanistic level, we continue to gain an appreciation for the intimate relationship between mobile elements and their hosts, and how mobile elements have adapted and responded to their hosts' cellular environment and regulatory pathways. Our research also has impact beyond our community, due to the many uses mobile elements provide as vectors for gene delivery and as mutagens that enable gene discovery.

The mobile DNA community has a strong appreciation and respect for biological inquiry at multiple levels. This view has been encouraged since the rediscovery of mobile genetic elements in the late 1970's. For example, the mobile DNA community holds international meetings every few years that are characterized by sessions ranging from molecular mechanisms to evolutionary impacts of mobile genetic elements. Despite growth of the discipline, the breadth of these international meetings has persisted because of what has been gained from this broad perspective. One goal of this journal is to propagate and reinforce our discipline's appreciation for breadth of biological inquiry.

The theme of breadth and inclusivity has also characterized the published volumes compiled by mobile DNA researchers. For example, in 1983, James Shapiro edited a volume of articles entitled *Mobile Genetic Elements *[1], which was followed by *Mobile DNA *in 1989 [2] and *Mobile DNA II *in 2002 [3]. *Mobile DNA II *has become an essential reference book for many laboratories, despite its awkwardly large size - the consequence of both the rapid growth of the discipline and the community's adherence to the theme of inclusivity. We hope that this journal - *Mobile DNA *- will provide a new forum for coalescing the community's most recent scientific discoveries.

As an online, open access journal, all articles published in *Mobile DNA *are available to readers without charge [4]. We hope that this will ensure that our collective findings are available to all, and that the stories of our discipline are not limited to what can be contained in a single volume. Further, we hope that the journal will serve as a forum to discuss new directions for research as well as to reflect upon what we've learned by recounting past accomplishments in our discipline. In this regard, we are pleased that our inaugural edition contains a review by James Shapiro on mobile DNA and evolution, some 27 years after he edited his important book *Mobile Genetic Elements*.

The editors of *Mobile DNA *hope the journal will become the preferred venue for the publication of new insights into all types of DNA rearrangements, ranging from transposition and other specialized recombination mechanisms to patterns and processes of mobile element and host genome evolution. We welcome submissions on the mechanisms of recombination using genetic, biochemical and structural approaches, regulation of DNA rearrangements by the environment and during development, and how DNA rearrangements affect cellular function and organism evolution. Manuscripts describing DNA rearrangements such as copy number variation and the application of new mobile element technologies are also encouraged.

The following article types will be considered: research articles reporting data from original research; comprehensive, authoritative review articles of any subject within the journal's scope; and methodology articles that present new experimental methods, tests or procedures. As an online journal, there are no constraints on the number of color figures and movies, or the size of datasets, that can be included in manuscripts.

Manuscripts submitted to *Mobile DNA *will be reviewed by internationally recognized experts in the field of mobile genetic elements, selected in part from our extensive editorial board [5]. The review process will be rapid, and the suitability of a manuscript for publication will be assessed solely on criteria of scientific excellence. To expedite the publication process, accepted manuscripts are made available primarily as provisional PDF files, then subsequently published in both fully browseable web form and as a formatted PDF.

*Mobile DNA *is the only journal solely dedicated to our discipline, and we hope you share our vision for what can be gained by launching this new forum for the international community of mobile DNA researchers to communicate. The editors are committed to making *Mobile DNA *a success, and we welcome your input as we begin this venture.

## Competing interests

The authors declare that they have no competing interests.
